# Living with Duchenne Muscular Dystrophy Beyond the Physical Implications: Cognitive Features, Psychopathology Aspects, and Psychosocial Resources—A Narrative Review

**DOI:** 10.3390/brainsci15070695

**Published:** 2025-06-28

**Authors:** Federica Tizzoni, Giulia Canella, Antonella Delle Fave, Daniele Di Lernia, Maria Luisa Lorusso, Maria Nobile, Maria Grazia D’Angelo

**Affiliations:** 1 Scientific Institute, IRCCS E. Medea, Unit of Child Psychopathology, 23842 Bosisio Parini, LC, Italy; federica.tizzoni@lanostrafamiglia.it (F.T.); marialuisa.lorusso@lanostrafamiglia.it (M.L.L.); 2 Scientific Institute, IRCCS E. Medea, Unit of Rare Diseases of the Central and Peripheral Nervous System, 23842 Bosisio Parini, LC, Italy; giulia.canella@lanostrafamiglia.it (G.C.); grazia.dangelo@lanostrafamiglia.it (M.G.D.); 3 Department of Pathophysiology and Transplantation, University of Milano, 20122 Milan, MI, Italy; antonella.dellefave@unimi.it; 4 Department of Theoretical and Applied Sciences, e-Campus University, 22060 Novedrate, CO, Italy; daniele.dilernia@uniecampus.it

**Keywords:** Duchenne muscular dystrophy (DMD), cognitive functioning, psychopathology, psychosocial resources, well-being, quality of life, neurodevelopmental disorders

## Abstract

**Background/Objectives**: Duchenne muscular dystrophy (DMD) is often discussed in the literature with regard to physical impairments. This narrative review aims to show that living with DMD involves psychological, psychosocial, and cognitive aspects in addition to the well-known physical complications. **Methods**: Firstly, this review examines the main cognitive functions affecting subjects with DMD and the possible role of dystrophin gene mutations on the central nervous system. Secondly, it analyzes the comorbidity between DMD, neurodevelopmental disorders (autism spectrum disorders, attention-deficit/hyperactivity disorder, obsessive–compulsive disorder) and psychopathological traits (anxiety and/or depressive symptoms). Finally, the review addresses the relatively sparse literature investigating the positive aspects associated with the experience of DMD, like psychosocial resources, resilience, subjective well-being, positive individual and social functioning, and social support. **Results**: DMD has a significant impact on cognitive areas, probably due to dystrophin deficiency in the brain. The prevalence of neurodevelopmental comorbidities and psychopathological symptoms is also higher in people with DMD than in the general population. Despite these challenges, emerging studies highlight the role of psychosocial and environmental resources, including resilience and supportive social relations, in promoting a good quality of life and successful adaptation to disease progression. **Conclusions**: Early recognition of the above difficulties and strengths could ensure better care and promote an overall better quality of life for people with DMD and their families, physically, psychologically, and socially. Preclinical and clinical research is moving in the direction of finding new therapies, treatments, and psychosocial interventions to pursue these goals.

## 1. Introduction

Duchenne muscular dystrophy (DMD) is a progressive, X-linked inherited disease caused by mutations in the dystrophin gene. These mutations lead to the absence or deficiency of the dystrophin protein, which is responsible for maintaining the integrity of muscle cells, resulting in the continuous degeneration of muscle fibers. It is characterized by progressive muscular weakness, cardiomyopathy, and respiratory failure. DMD has an estimated worldwide incidence of 1/3300 births, affecting males and, in rare cases, females, who usually exhibit a milder phenotype. The earliest symptoms, presenting at around 2–3 years of age, are usually difficulties with maintaining balance, climbing stairs, a waddling gait, and frequent falls. Most people with DMD become wheelchair-dependent at around 10–12 years of age and need assisted ventilation at around 20 years of age [1]. Due to advances in medical care, including improved respiratory and cardiac management and steroid therapy, survival rates have increased, and many individuals with DMD now live into their 30s or beyond [1,2,3,4]. These improvements in care present new demands. On the one hand, there is the management of typical adulthood issues, such as independence, leaving the family of origin and the management of new health problems (physical and psychological); on the other hand, there is also the necessary acceptance of the fact that, despite the progress in therapeutic research, at the moment, the majority of elderly individuals with DMD (or simply those confined to wheelchairs) do not have access or have very few possibilities of accessing experimental therapies or innovative treatments. As previously defined, DMD is caused by mutations in the DMD gene, a huge gene containing 79 exons and 8 promoters that is expressed in skeletal, cardiac, smooth muscle tissues and also in the human central nervous system. In the central nervous system, the full-length dystrophin isoform is expressed (Dp427). The dystrophin gene has at least four internal promoters, giving rise to shorter dystrophin products (Dp260, Dp140, Dp116, and Dp71) [5,6]. DMD gene mutations result in a loss of the full-length dystrophin protein (Dp427). Dystrophin Dp427 plays a critical role in maintaining the structure and stability of muscle and nerve cells, and it is altered in all individuals with DMD. There are three major isoforms of Dp427: Dp427-M (muscle), expressed mainly in skeletal and cardiac muscle; Dp427-B (brain), expressed in various areas of the central nervous system; Dp427-P (Purkinje), expressed mainly in Purkinje cells of the cerebellum. The Dp427B isoform and two short brain isoforms expressed by internal promoters, Dp140 and Dp71, are thought to be expressed throughout the cerebral cortex with the highest expression in the temporal and frontal cortex, the amygdala, and the hippocampus [7,8]. These regions are important in central nervous system development, neuroplasticity, and synaptogenesis, and them alterations can lead to an increased risk of developing cognitive, neuropsychological and/or neuropsychiatric disorders [9,10]. The hippocampus and Purkinje cell structures are part of a network that connects with the frontal lobe, which plays an important role in human memory and is responsible for executive functions, such as decision-making skills, judgment, attention span, and inhibition [5]. An important role is played by the position of the gene’s mutation: mutations near the beginning of the gene will affect only the longest isoforms, which are expressed mainly in skeletal and cardiac muscle; mutations progressively further along the gene (downstream of exon 44 and onwards) affect other isoforms that are expressed particularly in the brain, such as Dp140 and Dp71 [11]. As observed in several studies, mutations in the dystrophin gene, particularly in the distal portion, are often associated with specific cognitive deficits or neurodevelopmental disorders [12,13]. Altered synaptic function [14], alterations in the blood–brain barrier [15], neuroinflammation [16], altered calcium homeostasis and, more generally, altered ionic homeostasis and ion channel function [17,18] may all contribute to the neurological manifestation of DMD, but further researches are needed. While the neuropsychological and psychological aspects of individuals with DMD may have negative consequences for them and their families, resilience and well-being may play a crucial role in helping them cope with daily difficulties. However, there is limited evidence on the positive factors contributing to mental health, quality of life, and effective adaptation strategies among those living with DMD. For this reason, this narrative review aims to highlight the cognitive, psychological, and psychosocial resources present in individuals with DMD, which are not always considered.

To achieve our aim, we searched PubMed from 2000 to 2025 to analyze the most recent literature corresponding with our focus. We excluded papers that were not written in English, that dealt with other diagnoses, or that were off-topic.

## 2. Cognitive Abilities

In 1861, describing the first clinical case of a boy with muscular dystrophy, Duchenne used the definition of “caractere obtus”, effectively suggesting a cognitive impairment.

Throughout history, the main focus in DMD has been on muscle impairment, neglecting the cognitive manifestations of the disease [19].

Only in the last 20 years has it been possible to recognize DMD as a condition with significant neurological and cognitive implications. This has led to more attention being paid to the cognitive profile of these individuals, suggesting that cognitive impairment is non-progressive and not related to the severity of the muscle disease [20].

Dystrophin isoforms expressed in the central nervous system, particularly Dp140 and Dp71, play a relevant role in the brain and in the development of cognitive abilities in individuals with DMD [21]. The Dp140, encoded by an internal promoter of the DMD gene located in intron 44, is expressed primarily in the central nervous system, particularly during fetal brain development. Its amount decreases in the adult brain.

Individuals with mutations downstream of exon 44 are more likely to experience problems with fine motor and visual perceptual skills, speech, and probably some locomotor aspects. This suggests a role for brain dystrophin isoforms in the development of coordination and motor dysfunction [22]. Research conducted by Pane and colleagues found that younger subjects, assessed with the Griffiths scale, had a lower developmental quotient (DQ) and had difficulties in hearing, speech and in performance tasks that older individuals with similar gene mutations were able to perform. This suggests that these difficulties were not mutation-dependent, but rather age-dependent, suggesting a delay in developmental milestones rather than general disability and a longer latency in acquiring those specific skills [22]. Several authors showed a close association between the increased risk of cognitive impairment and deletions in the distal portion of the DMD gene (ranging from exon 45 to exon 60), specifically those involving the Dp140 isoform [23,24]. In particular, children with DMD who do not express Dp140 tend to have a lower intelligence quotient (IQ) and greater cognitive difficulties than those who retain this isoform [25]. Some authors reported that individuals lacking the Dp140 isoform show deficits in verbal working memory and processing speed [26,27]. The biochemical and tissue characteristics of Dp140 suggest that this isoform plays an important function in normal cognitive development, highlighting a potential association between the preservation of these regulatory sequences and the maintenance of normal cognitive function in people with DMD [24]. Dp71 is the shortest isoform and is encoded by a promoter far downstream, near the 3′ end of the DMD gene (starting at exon 63). It is implicated in multiple cellular processes, and it gradually increases from the embryonic stage to the adult stage. Several studies describe mutations in these isoforms as a factor contributing to the severity of cognitive impairment [28,29]. The absence or reduction of these isoforms has been linked to a distinct cognitive profile observed in individuals with DMD.

In individuals with mutations affecting all distal isoforms, there is a strong association with severe learning disability and an IQ that is two standard deviations (SD) lower than normal; in individuals with mutations affecting only those isoforms upstream of exon 30, a minimal frequency of intellectual impairment is observed [11].

A recent meta-analysis has investigated the cognitive profile of individuals with DMD, highlighting that the mean full-scale IQ is approximately one SD below the average norm [30]. In another review, intellectual disability in individuals with DMD was observed in 22% of the patients, with a lower prevalence in patients with the exclusive involvement of Dp427 and higher rates in those with additional Dp140 or Dp71 involvement [9]. In line with this result, D’Angelo and colleagues showed that children with DMD have an average IQ that is about one SD lower than the population average. They also found a discrepancy between the verbal intelligence quotient (VIQ) and the performance intelligence quotient (PIQ), showing that VIQ is more affected than PIQ [31]. The above discrepancy is well established and broadly acknowledged in the current literature [30].

These difficulties in verbal competence seem to be associated with specific neuropsychological deficits; children with DMD may have difficulties in the maintenance and manipulation of verbal information, immediate verbal memory, verbal working memory, verbal comprehension, vocabulary, and verbal learning and encoding.

A study conducted by Lo Russo and colleagues showed that verbal deficits tend to be more evident in receptive speech than in expressive language [32]. Poor verbal skills seem to play a role in learning abilities. These children may have problems with processing large amounts of information; for example, when following a lesson in the classroom. As a result, they would benefit from breaking information and tasks into concise and simple steps [33]. Furthermore, Lo Russo’s study showed the presence of reading difficulties in boys with DMD, essentially involving speed parameters [32]. The type of language spoken by the individuals plays an important role: in orthographically transparent languages, such as Italian, the letter-to-sound conversion is relatively intact but, for example, in French, individuals have greater reading disabilities because of their opaque language.

It has been observed in the literature that reading disabilities and reduced arithmetic skills are prevalent in individuals with DMD, particularly in those with mutations affecting both Dp140 and Dp71 [34].

Despite normal IQ scores in some individuals, deficits in working memory and executive functions, including inhibition, switching, problem-solving, and planning, are commonly reported [34,35].

**Figure 1:**
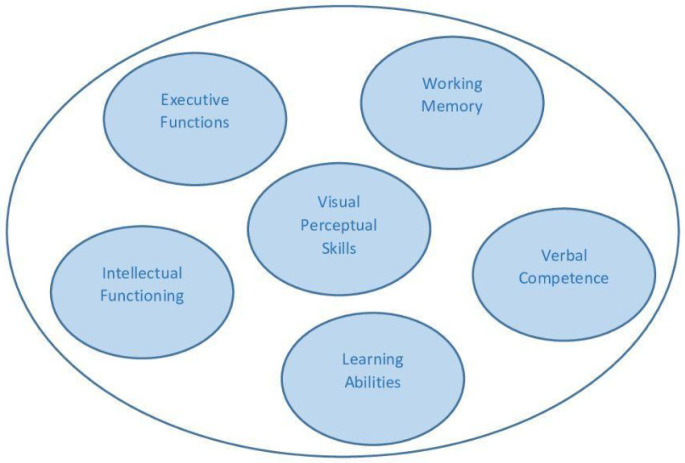
Main cognitive abilities affected in DMD.

The main cognitive domains that could be affected in individuals with DMD are illustrated in Figure 1.

The disruption of the dystrophin gene has an impact on brain functions, but the specific relationship between the loss of dystrophin isoforms in the brain and mental functioning has not been clarified yet.

Significant neurobiological advances in understanding the role of dystrophins in the brain have largely stemmed from studies using dystrophic mouse models [36]. The mdx mouse, which lacks the Dp427 isoform, demonstrates specific deficits in learning and memory, as well as marked abnormalities in emotional and social behaviors. One of the most notable features of this model is an increased sensitivity to stress, often expressed as exaggerated unconditioned fear responses to mild stimuli. This phenotype has been associated with amygdala dysfunction and reduced GABAergic inhibition [37]. Such heightened stress reactivity is consistently observed across various dystrophic animal models and mirrors the increased emotional vulnerability frequently reported in people with DMD [38,39,40]. However, while some of the behavioral traits observed in mice are clearly recognizable in affected individuals, the expression of these traits in humans is more heterogeneous, with considerable variability in their severity and prevalence [36].

Brain morphology and metabolic studies have not reveal structural abnormalities but they show specific alterations in people with DMD compared with healthy controls, such as total brain volume and cerebral gray-matter atrophy. Furthermore, reduced gray-matter volume and cerebral blood flow were observed in children lacking both Dp427 and Dp140 [41,42]. 

Abnormal dendrite development, myelin damage, or altered structural/functional connectivity are assumed to play a role in altered brain development in subjects with DMD [8]. Most studies on brain alterations in DMD focus on the correlation between brain abnormalities and the clinical phenotype and compare subgroups of patients based on mutations.

Doorenweerd and colleagues showed through pseudo-continuous arterial spin-labeling MRI that there was lower cerebral blood flow (CBF) in DMD subjects than in control subjects; moreover, DMD individuals lacking Dp140 had a lower CBF than the ones with preserved Dp140 [43].

Recent studies using diffusion tensor imaging (DTI) have revealed elevated diffusivities in the prefrontal cortex of individuals with DMD. This finding is accompanied by increased taurine levels in the same cerebral area and a decrease in fractional anisotropy in the hippocampus, along with metabolic changes. These changes suggest potential impairments in axonal and myelin integrity, as well as reduced neuronal density in the white matter [44,45,46].

Most previous studies focused on the brain of DMD patients in comparison with healthy controls and did not account for possible comorbidities such as cognitive impairment and/or neurodevelopmental disorders.

Given the complex comorbidities in DMD, at our institute, Peruzzo et al. recently used a multimodal, multivariate approach to analyze DMD-related effects in the central nervous system (CNS) to identify a pattern of brain structural alterations uniquely associated with the disease. A group of people with DMD were compared with a control group that included not only healthy people but also participants diagnosed with cognitive impairment and autism spectrum disorder (ASD) without DMD gene alterations. Through this method of analysis, we were able to show that white-matter alterations, particularly of long fiber bundles, were selectively observed in individuals with DMD, suggesting the presence of a specific dystrophin-related pattern [47].

Recent therapeutic advances have made it clear how important early diagnosis and evaluations are. A detailed assessment as early as possible would identify the child’s difficulties and suggest the most appropriate intervention.

Given the non-progressive nature of cognitive deficits in DMD, early recognition and intervention are critical for optimizing developmental outcomes and improving the quality of life of affected individuals and their families.

### Pharmacological and Gene Therapy

In recent years, remarkable progress has been made in the therapeutic landscape for DMD, offering new strategies for disease modification and symptom control. Gene therapy is considered one of the most transformative of these developments. Delandistrogene moxeparvovec is the most widely adeno-associated-virus-delivered micro-dystrophin gene therapy. Despite its promise, recent reports of treatment-associated acute liver failure and fatalities have raised safety concerns, emphasizing the importance of strict patient monitoring and risk mitigation strategies [48].

Equally important are exon-skipping therapies with antisense oligonucleotides that restore a partially functioning dystrophin by bypassing specific mutations [49].

Despite their potential for the treatment of DMD, the effects of gene therapies and exon-skipping approaches on cognitive and neuropsychological functions are not yet fully understood.

Recently, interventional studies in mdx mouse models, such as antisense oligonucleotide therapy, have shown restoration of dystrophin in the mouse brain and partial improvement of cognitive impairment [50].

Pharmacological treatments aimed at modulating inflammation and fibrosis are also central to current care [51] and may play a role in cognitive function [17,52,53].

Lopez et al. reported that increasing endogenous nitric oxide levels restored calcium homeostasis, decreased reactive oxygen species production, and improved cognitive function in mdx mice [54].

These preliminary results seem to be very promising and open up a new era of intervention in DMD individuals that addresses not only muscle function but also “brain” function. However, we still need to be very cautious.

## 3. Psychopathological Aspects

As life expectancy has increased, more attention has been paid to the health-related quality of life of people with DMD, their psychological and psychosocial care, and their transition to adulthood. As a result, the goal of care has evolved from simply prolonging life to improving the quality of life, particularly in terms of mental health and psychosocial functioning [55,56].

Research showed that children and adults with DMD have a higher risk of developing symptoms of neurodevelopmental disorders and psychopathological traits than the general population. The most frequent ones are autism spectrum disorder (ASD), attention-deficit/hyperactivity disorder (ADHD), obsessive–compulsive disorder (OCD), and emotional and behavioral disorders, including depression, aggression, and anxiety [13,38,55].

Several studies have investigated the frequency of neurodevelopmental disorders and psychopathological traits, particularly internalizing traits, in individuals with DMD reporting similar prevalence rates. The variability in the rates probably reflects the different methods used, such as the age of the sample; for example, when the study takes into account the entire age range of DMD symptomatology, the measure of the frequency of these traits might be more accurate because the onset of symptoms can be spread over a large time span [13,38].

The prevalence of these disorders and traits in individuals with DMD appears to be higher than the incidence rate in the general population. Autism spectrum disorder (ASD) occurs in 3–20% of cases, in contrast to the general pediatric population, which is about 0.6% [5,13,38,55,57,58].

Attention-deficit hyperactivity disorder (ADHD) is one of the most observed psychiatric comorbidities among children with DMD, with 11–32% of observed cases, showing a higher rate than in the general population, which is about 6–7% [5,12].

The frequency of ADHD in DMD may vary due to several aspects, including the method used for screening or the different manifestations of symptomatology. For example, children with DMD may not show the same kind of symptoms of hyperactivity and agitation. It is important to note that in children with DMD, these behaviors may be less noticeable due to their motor problems, muscle weakness, and physical limitations, and therefore, measures of physical movement may not be positively evaluated. It is equally important to consider that some of the cognitive patterns observed in DMD, such as short-term memory and attention problems, or behavioral problems resulting from the side effects of steroid therapy may lead to a misdiagnosis of ADHD in the child [12,59].

Evidence shows that the incidence of obsessive–compulsive disorder (OCD) in individuals with DMD is around 5–11%, with higher prevalence than in the general population, which is about 2–3%. Boys with DMD frequently have a recognizable obsessive compulsive phenotype with associated anxiety [5,60].

Finally, features consistent with anxiety have been found in 24–29% of individuals with DMD, a higher rate compared with the 10–30% estimated in the general pediatric population. Depressive symptoms are evident in 17–27% [5,13,38,55,58,60]. Young children may develop emotional disorders that manifest as oppositional behaviors or anger outbursts rather than the classic clinical presentation of depression or anxiety. In contrast, regarding externalizing aspects, there are no clear and uniform data from the literature.

Most studies on the psychopathology of individuals with DMD rely on clinical assessments or parent-reported questionnaires. This may result in a difference in the assessment of symptomatology from what is actually perceived by the children themselves. These discrepancies may be explained by differences between the home environment, in which the parent sees their child, and the actual experience of the child when they are alone; differences in the attribution of their behavior—for example, due to temperament or derived from an emotionally charged situation; and by the person’s psychopathology. It is noteworthy that the self-perception of many children and adolescents with DMD is more positive than what their caregivers describe [58,59].

Regarding the risk of developing comorbidities with neurodevelopmental disorders and behavioral and emotional difficulties, the literature suggests a possible correlation with genetic mutations, indicating a role for Dp427, Dp140, and Dp71 [12,13,61]. It appears that individuals with mutations that affect multiple brain isoforms or with complete and shorter brain isoforms have more severe cognitive and behavioral problems [7].

Individuals with mutations downstream of exon 30 appear to have a higher risk of developing these comorbidities than those whose mutation affects only the longer isoform of Dp427 [13,38]. It is important to underline that the isoforms associated with a higher risk of developing comorbidities with neurodevelopmental disorders are the same ones associated with a higher risk of cognitive impairment; that is, Dp71 and Dp140.

Therefore, the increased risk of ADHD, ASD, anxiety, and OCD in boys with DMD may be related to the lack of dystrophin expression in the brain or to the effect of dystrophin on other central nervous system proteins [5]. In contrast to neurodevelopmental features, behavioral and emotional features such as obsessive/compulsive features, anxiety, and depressed mood appear to be well distributed among the genotype subgroups. The relatively uniform distribution of these symptoms suggests that the pathogenic mechanisms underlying emotional and behavioral symptoms may differ from those responsible for neurodevelopmental symptoms. Indeed, it is conceivable that mutations that alter the expression of longer dystrophin isoforms—including those located far upstream, near the 5′ end of the dystrophin gene—contribute to the development of these symptoms [38].

Instead, studies investigating the association between ASD and genetics have provided mixed results. Some of these studies have demonstrated possible alterations in a specific region of the dystrophin gene, located near but not within it; for example, mutations that interrupt the Dp140 isoform of dystrophin. This would explain the coexistence of double diagnoses in the same patient [5,62,63]. If this transcript is found in the muscle, it gives rise to DMD, whereas if it is found in the central nervous system, it gives rise to neuronal dysfunction, with the possible consequence of ASD [62].

In contrast, the meta-analysis conducted by Pascual-Morena’s research group suggests that the Dp71 isoform modulates the risk of developmental disorders in DMD. Although the meta-analysis did not show a statistically significant association for ASD, a clear trend toward a deleterious association in DMD was observed when Dp71 was involved. From their work, it appears that Dp140 has no influence on ASD [61]. Although the association between dystrophin isoforms and ASD remains unclear, these studies demonstrate a potential genetic link between DMD and ASD.

On the other hand, evidence of the association between ADHD and possible genetic mutations seems to be more consistent. Research suggests the possible involvement of Dp140 and Dp71 isoforms, although the study by Pascual-Morena et al. seems to show a less significant association with Dp140 [12,61].

Studies have shown that ADHD is more frequently associated with mutations affecting the expression of Dp140 (mutations in exons 45–55) and mutations affecting all short isoforms of dystrophin, including Dp71 (mutations in exons 62 and 63). Therefore, a higher frequency is observed in individuals with mutations affecting the expression of dystrophin in the brain [5,12,63]. However, other genetic and/or environmental modifiers may play a role in determining the development, phenotype severity, and phenotypic spectrum of the neurocognitive profile in ADHD. Sometimes patients with identical mutations do not exhibit the same cognitive or behavioral phenotype [5,12,63]. Regarding emotional-related disorders, the research does not suggest an association between the isoforms involved and the prevalence risk of emotional and behavioral dysregulation, depression, anxiety disorders, and OCD [61]. It cannot be ruled out that mood disorders could also develop as a result of becoming aware of the disease, and living with the disease itself could also be a risk factor for the onset of internalizing traits.

However, the association between neuropsychiatric disorders and the number of dystrophin isoforms involved, summarized in Table 1, is not distinctly clear. Therefore, further research is needed.

Given the high incidence of psychiatric or neurodevelopmental comorbidities, it is important for people with DMD to be evaluated by a mental health professional so that necessary and more appropriate treatments can be identified. Mild symptoms may benefit from psychosocial and/or psychotherapeutic interventions. If the severity of symptoms is greater and, consequently, this type of intervention is not sufficient and effective, psychopharmacological treatment may be necessary [56]. However, research studies have highlighted a significant gap in the knowledge of psychopharmacological treatments for mental disorders in people with neuromuscular diseases. Therefore, the overall low level of evidence from published data does not allow for the formulation of recommendations that clinicians can rely upon [56].

Steroid therapy is the gold-standard therapy for stabilizing muscle strength, extending walking and standing abilities, and supporting respiratory function in people with DMD [3,4]; nevertheless, boys taking steroids show more externalizing behavioral problems, i.e., aggressive behaviors, than boys not taking steroids [7,12]. The impact of corticosteroids on behavior remains controversial; long-term exposure may be associated with initial mood swings, as well as memory and attention difficulties, which may stabilize over time [5,12]. Effects of medications usually prescribed by first-line providers to manage acute and chronic medical conditions may worsen psychiatric symptoms in DMD individuals [55,56].

The use of psychotropic drugs for psychiatric manifestations in people with neuromuscular diseases may be hindered by multiple factors, including the ability to monitor their efficacy and safety and the risk exposure to multisystem complications, such as weight gain or the occurrence of heart disease. The latter may represent a contraindication to the administration of antidepressant, atypical antipsychotic, or CNS stimulant psychotropic drugs [55,56].

Despite the possible presence of psychiatric and neurodevelopmental comorbidities, people with DMD and their families may use specific coping strategies that help them deal with illness and stressful situations and, thus, make their emotional and behavioral difficulties milder [59].

Evidence suggests that most children with DMD cope relatively well with the disorder; in particular, overall adjustment improves as they get older [5,59,64]. The term “psychosocial adjustment” is used in reference to emotional, behavioral, and social functioning; it is a central aspect of quality of life and provides a good estimate of how an individual copes with DMD [64].

However, relationships with peers become more problematic with advancing age, as declining physical functioning and health may result in reduced access to social and recreational opportunities. In fact, children with DMD exhibit unexpectedly high levels of social and communication difficulties, regardless of a diagnosis of ASD. Other difficulties are observed in children with ADHD comorbidity, who report significantly worse psychosocial adjustment than children with DMD without an ADHD diagnosis. Behaviors commonly associated with ADHD, including poor emotional regulation, low frustration tolerance, aggression, oppositional/argumentative behavior, and mental rigidity/inflexibility, can be significantly problematic and stressful for families with DMD [5,64].

Symptoms of anxiety and depression affect quality of life and are often associated with poor adjustment and coping skills [60]. Personal resources of people with DMD and their family members may moderate the relationship between illness and psychological adjustment. Caregivers adopting maladaptive coping strategies, such as avoidance, report higher levels of stress, distress, frequent feelings of guilt, low self-esteem, sadness, and depression related to their care recipient’s condition. In contrast, caregivers showing high self-esteem, a sense of support, and a positive perception of their own experience tend to have better coping skills, accepting reality rather than denying it, and positively reinterpreting the experience. This could lead to better long-term emotional regulation, with a positive influence on the care recipient’s emotional well-being and stress levels. Therefore, caregivers’ use of avoidant coping strategies is associated with an increased risk of emotional/behavioral problems in individuals with DMD [65].

## 4. Psychosocial Resources and Well-Being of People with DMD

As documented in the previous sections, problematic cognitive, neuropsychological, and psychopathological aspects of DMD and their negative consequences on individual and family life were broadly investigated. Limited attention was paid to the exploration of positive indicators of mental health, quality of life, and successful adaptation among people with DMD. Their psychological functioning is primarily assessed through measures of depression, anxiety, and psychopathology, whilst quality of life is predominantly investigated in terms of health-related limitations. More generally, a biomedical and pathogenic approach still dominates in scientific investigations of the experiences reported by individuals living with chronic conditions, such as DMD, that severely compromise their health and daily functioning [66]. Consequently, these people are unilaterally viewed through the lens of impairments, disability, and vulnerability, a priori excluding the presence of positive dimensions of well-being in their lives. This approach openly contradicts the definition of health proposed by the World Health Organization (WHO)—which has not been amended since 1946—as a state of complete physical, mental, and social well-being, rather than as an absence of ill-being.

During the last three decades, a variety of studies have shown that personal and relational resources are indeed available and play a substantial role in supporting and promoting well-being, positive mental health, and a good quality of life among people diagnosed with chronic and progressive diseases [67,68,69,70,71]. In this section, we therefore review the literature concerning quality of life (QoL), health-related quality of life (HRQoL), well-being, and psychosocial resources among people diagnosed with DMD. We adopt a holistic perspective that recognizes well-being and, by extension, global quality of life as dimensions nurtured by the presence of positive aspects, rather than by the mere absence of symptoms. Consistent with the vast amount of literature addressing this issue in the context of different chronic conditions [72,73,74,75], we assume the co-existence of psychological ill-being with positive dimensions such as resilience, emotional balance, and social support that contribute meaningfully to QoL and positive mental health in people with DMD. Several studies support this view, based on data collected through different methodologies and from among participants differing in disease progression stage and cultural context. Related findings challenge traditional assumptions about the prominent association of physical suffering with poor mental health.

The first recurrent evidence is the overall preservation of psychosocial and emotional well-being despite the progressive physical limitations entailed by the disease. Although, as expected, lower levels of HRQoL were reported by people with DMD compared with the general population [76], findings about global QoL and psychosocial functioning are much more articulated. Not surprisingly, physical domains consistently showed the most significant impairments, and strong correlations were detected between motor function and physical health. Chronic pain was associated with reduced physical functioning and lower QoL across all domains [77]. At the same time, broad variations in the relationship between physical deterioration on psychosocial functioning were detected across studies. The correlations between motor abilities and psychosocial/social functioning were only moderate [78]. Clinically relevant affective disorders (anxiety and mild depression) emerged in a minority of participants both in studies involving adolescents [77] and adults [79]. An age-related decline in social engagement was observed in adolescents with DMD compared with age-matched controls, but it was uncorrelated with QoL scores [80]. Some studies detected impaired psychosocial HRQoL among children and adolescents, but they were limited in number and based on assessment measures grounded in the view of the disease as the primary determinant of individual well-being [78,81,82,83].

An increasing number of studies instead documented good levels of global quality of life (QoL) and average levels of mental and emotional well-being among people with DMD across ages and disease progression stages [84,85,86,87,88,89]. Overall, the findings suggest that QoL does not necessarily deteriorate with disease progression. A significant decrease in the correlation between QoL scores and functional measures of walking and fatigue was detected over one year among ambulatory children [90]. Stable QoL scores were detected over nine months across age groups, with no significant differences between younger and older participants, and even with improvements in the domains of personal development, personal satisfaction, leisure/recreation, and interpersonal relationships [91]. Good psychosocial HRQoL and stable low depression scores across age groups [80] as well as a positive association detected between age and HRQoL [92] supported the potential for people with DMD to successfully adapt to disease progression. Stability of psychosocial functioning levels was detected in the face of increasing limitations in physical functioning, such as wheelchair-dependency [85,93] and the need for mechanical ventilation [94]. In a study involving children and adolescents, clinical disease progression explained a lower percentage of variance in HRQoL compared with environmental and social factors, which played a more relevant role as determinants of HRQoL levels [92]. Finally, the joint assessment of both HRQoL and global QoL among children with DMD and their parents highlighted that the two constructs are clearly distinct, though partially correlated [95]. In particular, children’s ratings of HRQoL explained only 21% of the global QoL variance.

Studies comparing people with DMD and healthy participants further supported this evidence. Children and adolescents with DMD reported lower HRQoL scores in physical and social functioning compared with their healthy peers, but no significant group difference emerged in emotional functioning levels [96]. A lower quality of life was reported by adolescents with DMD in the domains of physical health and friend networks, but the scores of all the other quality of life areas were similar to healthy peers’ ones [85,97].

Psychological and social resources, in particular resilience and social support, emerged as relevant assets in promoting well-being among people with DMD. In studies conducted among adults, resilience—defined as the dynamic process of individuals’ satisfactory adaptation to adversarial conditions—was negatively correlated with psychopathological symptoms among adults [79]. Similarly, psychological flexibility—closely related to resilience—was associated with high levels of life satisfaction [98]. Studies involving adolescents and youth showed that positive parental adjustment significantly predicted resilient behavior in the children independently of clinical severity measures [84].

Findings concerning social support from family and significant others provide a more complex picture. Social support was associated with normal psychosocial functioning among youth [84], but it was unrelated to psychopathological symptoms among adults with DMD [79]. A specific age-related decline was detected in social relationships among adolescents and young adults [83] and among boys older than 10 compared with younger children [80]. Notably, the transition from childhood to adolescence (ages 8–12) was recurrently identified as a particularly vulnerable period, in which participants with DMD reported significantly lower HRQoL, most likely related to the progressive loss of autonomous walking compared with peers with other chronic conditions [99].

Lastly, a recurrent discrepancy in perceived well-being and QoL– well documented by a vast amount of literature spanning decades—was detected between people with DMD and their caregivers. Parents consistently rated the overall HRQoL of their sons with DMD as significantly lower than the boys’ self-reports across various life domains, such as social functioning, moods/emotions, self-perception, and social acceptance [85], physical activities/health and general mood/feelings [59,96,97], social skills and functional communication [89]. Overall, these findings show that despite caregivers’ concerns, youth with DMD maintain more positive self-perceptions and confidence in their psychological and relational resources.

Overall, the research findings summarized in this section highlight a limited correlation of disease severity with psychosocial resources and individual quality of life. They suggest the need to overcome deficit-focused models by adopting a more comprehensive approach in the investigation of health and well-being in chronic conditions. Well-being and quality of life represent complex constructs that are not merely determined by physical health status; other objective and subjective dimensions, including personal, social, and environmental assets may effectively buffer the negative consequences of physical impairments. Empirical evidence from the reviewed studies reveals the complex intertwining between DMD-related limitations and the individuals’ preservation or development of psychological and relational resources that allow them to attain well-being and a good QoL. Assessment measures selectively focused on pathology and impairments may not adequately capture the complexity of the DMD experience. To achieve a comprehensive view, it is important to evaluate both problematic and constructive dimensions. As shown by empirical evidence, positive indicators of well-being, psychosocial resources such as resilience and social support, environmental assets, and facilitators effectively promote good mental health beyond symptom management.

### Psychological and Psychosocial Interventions

Several intervention models and programs are currently available to help people with DMD and their families [55].

Psychotherapeutic strategies, like cognitive–behavioral therapy (CBT) and acceptance and commitment therapy (ACT), may provide significant benefits, improving well-being and quality of life in adults with neuromuscular diseases [100].

These strategies could also be usefully implemented with youth with DMD and their families to improve their coping, self-regulation, and adjustment.

Psychoeducational interventions may also be beneficial to people with DMD and their families, helping them better understand their disease symptoms and manifestations, the impacts on their daily life, and treatment options.

Beyond structured psychotherapeutic and psychosocial interventions, hermeneutic–phenomenological methodologies allow for in-depth exploration of the lived experiences reported by affected individuals and their families, revealing existential and meaning-making dimensions of coping [101].

Incorporating such hermeneutic–phenomenological dimensions into clinical practice may enhance emotional well-being, enrich coping strategies, and improve the overall quality of life for individuals with DMD and their caregivers.

Despite this evidence, access to psychological support remains limited. Integrating psychological services into multidisciplinary DMD teams is therefore essential to ensure comprehensive and family-centered care.

## 5. Conclusions

This narrative review highlights how DMD is a multifaceted condition that includes progressive muscle degeneration, which is now well documented, and cognitive, psychopathological, and psychosocial aspects. These latter aspects are less known but equally crucial, as they have a profound impact on the quality of life of people with DMD and their families. 

Genetic alterations responsible for the loss of specific dystrophin isoforms play a key role in the development of cognitive and neuropsychological deficits, such as IQ, working memory, language, executive functions, and academic skills. In contrast, the link between specific genetic mutations and psychopathological aspects is only partially understood. On the one hand, the presence of neurodevelopmental disorders seems to be more related to the lack of specific dystrophin isoforms; on the other hand, emotional and behavioral symptoms may be the result of a more multifactorial interaction between biological, psychological, coping strategies and environmental factors.

This dual mechanism of genetic susceptibility and environmental influences emphasizes the need for comprehensive and multidisciplinary care strategies.

Recently available treatments for DMD include pharmacological and genetic therapies that aim to modify dystrophin levels in the central nervous system. To date, the impact that these therapies could have on the cognitive abilities and behavior of these individuals is still not fully known.

As research develops new insights into the effects of gene therapies, it is important to consider several aspects when planning targeted educational and therapeutic interventions. These include deficits in verbal processing and memory, as they hinder academic performance and adaptive behavior, and cognitive impairment that does not progress over time, suggesting a developmental origin, as this may impact daily functioning.

At the same time, we emphasize the importance of psychological and social resources in promoting well-being despite the challenges of DMD. Resilience and psychological flexibility together with social support from family, friends, and community were found to be key protective factors for maintaining a satisfactory quality of life. It is of particular significance that, in many cases, the quality of life reported by people with DMD is higher than that reported by their caregivers.

This discrepancy underscores the need to listen to and to incorporate the perspective of those affected into care planning by recognizing their capacity for positive adaptation and their internal resources. An approach focused only on pathological aspects would not adequately capture the complexity of the experience lived by people with DMD. For this reason, it is important to develop interventions that also take into account positive dimensions such as psychological well-being, social relationships, and adaptability. A holistic approach that takes into account both the challenges and strengths of patients and their families as well as neuropsychological, psychopathological, and psychosocial aspects, together with early diagnosis, allows for the development of more targeted and personalized interventions. This allows for an increase in the quality of life and general well-being of patients and their families and opens up new perspectives for research and clinical practice.

## Figures and Tables

**Table 1:** Overview of psychopathological features, neurodevelopmental disorders, and genetics.

Neurodevelopmental Disorders and Psychopathology	Features	Description	References
Neurodevelopmental disorders (ASD, ADHD) and psychopathological traits (OCD, emotional/behavioral disorders)	Prevalence	Higher risk of disorders than the general population	[13,38,55]
	Genetics	Possible correlation with genetic mutations, especially a role for Dp427, Dp140, and Dp71. Mutations after exon 30 → increased risk of comorbidities	[12,61] [13,38]
Autism spectrum disorder (ASD)	Prevalence	3–20% in DMD vs. 0.6% in the general population	[5,13,38,55,57,58]
	Genetics	Mixed results, unclear: possible alteration of a specific region of the dystrophin gene; for example, Dp140; Dp71 potentially implicated	[5,62,63] [61]
Attention-deficit/hyperactivity disorder (ADHD)	Prevalence	11–32% in DMD vs. 6–7% general population	[5,12]
	Genetics	Possible involvement of mutations affecting Dp140 (exons 45–55) and Dp71 (exons 62 and 63)	[12,61]
Obsessive–compulsive disorder (OCD)	Prevalence	5–11% in DMD vs. 2–3% general population	[5,60]
	Genetics	No association with the isoforms	[61]
Anxiety and depression	Prevalence	Anxiety: 24–29% vs. 10–30% in the general population; Depression: 17–27%	[5,13,38,55,58,60]
	Genetics	Mutations that impact the expression of longer dystrophin isoforms, including those very far upstream near the 5′ end of the dystrophin gene	[38]

DMD: Duchenne muscular dystrophy; ASD: autism spectrum disorder; ADHD: attention-deficit/hyperactivity disorder; OCD: obsessive–compulsive disorder.

## Abbreviations

DMD	Duchenne Muscular Dystrophy
IQ	Intelligence Quotient
SD	Standard Deviation
VIQ	Verbal Intelligence Quotient
PIQ	Performance Intelligence Quotient
CBF	Cerebral Blood Flow
DTI	Diffusion Tensor Imaging
CNS	Central Nervous System
ASD	Autism Spectrum Disorder
ADHD	Attention-Deficit/Hyperactivity Disorder
OCD	Obsessive–Compulsive Disorder
WHO	World Health Organization
QoL	Quality of Life
HRQoL	Health-Related Quality of Life
CBT	Cognitive–Behavioral Therapy
ACT	Acceptance and Commitment Therapy
